# Hyperspectral Imaging Tera Hertz System for Soil Analysis: Initial Results

**DOI:** 10.3390/s20195660

**Published:** 2020-10-03

**Authors:** Volker Dworak, Benjamin Mahns, Jörn Selbeck, Robin Gebbers, Cornelia Weltzien

**Affiliations:** 1 Department Engineering for Crop Production, Leibniz-Institute for Agricultural Engineering and Bioeconomy (ATB), Max-Eyth-Allee 100, 14469 Potsdam, Germany; bmahns@atb-potsdam.de (B.M.); jselbeck@atb-potsdam.de (J.S.); rgebbers@atb-potsdam.de (R.G.); cweltzien@atb-potsdam.de (C.W.); 2 Faculty V of Mechanical Engineering and Transport Systems, Chair of Agromechatronics, Technische Universität Berlin, Strasse des 17. Juni 136, 10623 Berlin, Germany

**Keywords:** hyperspectral imaging, Mie scattering, soil imaging, soil sensing

## Abstract

Analyzing soils using conventional methods is often time consuming and costly due to their complexity. These methods require soil sampling (e.g., by augering), pretreatment of samples (e.g., sieving, extraction), and wet chemical analysis in the laboratory. Researchers are seeking alternative sensor-based methods that can provide immediate results with little or no excavation and pretreatment of samples. Currently, visible and infrared spectroscopy, electrical resistivity, gamma ray spectroscopy, and X-ray spectroscopy have been investigated extensively for their potential utility in soil sensing. Little research has been conducted on the application of THz (Tera Hertz) spectroscopy in soil science. The Tera Hertz band covers the frequency range between 100 GHz and 10 THz of the electromagnetic spectrum. One important feature of THz radiation is its correspondence with the particle size of the fine fraction of soil minerals (clay < 2 µm to sand < 2 mm). The particle size distribution is a fundamental soil property that governs soil water and nutrient content, among other characteristics. The interaction of THz radiation with soil particles creates detectable Mie scattering, which is the elastic scattering of electromagnetic waves by particles whose diameter corresponds approximately to the wavelength of the radiation. However, single-spot Mie scattering spectra are difficult to analyze and the understanding of interaction between THz radiation and soil material requires basic research. To improve the interpretation of THz spectra, a hyperspectral imaging system was developed. The addition of the spatial dimension to THz spectra helps to detect relevant features. Additionally, multiple samples can be scanned in parallel and measured under identical conditions, and the high number of data points within an image can improve the statistical accuracy. Technical details of the newly designed hyperspectral imaging THz system working from 250 to 370 GHz are provided. Results from measurements of different soil samples and buried objects in soil demonstrated its performance. The system achieved an optical resolution of about 2 mm. The sensitivity of signal damping to the changes in particle size of 100 µm is about 10 dB. Therefore, particle size variations in the µm range should be detectable. In conclusion, automated hyperspectral imaging reduced experimental effort and time consumption, and provided reliable results because of the measurement of hundreds of sample positions in one run. At this stage, the proposed setup cannot replace the current standard laboratory methods, but the present study represents the initial step to develop a new automated method for soil analysis and imaging.

## 1. Introduction

Soil is a fundamental resource in the earth’s ecosystems and for human life. The understanding of the soil’s status and function is highly relevant because soils contribute to the recycling, filtering, transformation, and buffering of substances, in addition to the production of food, forage, and biogenic raw materials. In particular, for sustainable agriculture soils must be analyzed regularly to assess their fertility. However, soil analysis with conventional sampling and laboratory-based methods is expensive and time consuming. Thus, scientists are searching for new, sensor-based methods that can analyze soils more efficiently. The development of soil sensors is a challenging task due to the complexity of the soil and its many interfering parameters. This is also true for proximal soil sensing [1,2]. To date, visible and infrared spectroscopy, electrical resistivity, gamma ray spectroscopy, and X-ray spectroscopy have been investigated extensively [2]. One versatile candidate sensor for performing nondestructive soil measurements is Tera Hertz (THz) spectroscopy, which covers the electromagnetic frequency range between 100 GHz and 10 THz [3]. An important feature of THz radiation is its correspondence with the particle size of the fine fraction of soil minerals (clay < 2 µm to sand < 2 mm). The particle size distribution is a fundamental soil property which governs soil water and nutrient content, among other characteristics. Another important feature of THz radiation is its ability to penetrate materials. Furthermore, it is nondestructive and not hazardous. However, little research has been conducted on the application of THz spectroscopy in soil science. Moreover, the development of THz systems is ongoing. Some non-continuous wave systems are used and the applications are in their infancy [4,5]. Even fewer studies have been performed on continuous wave (CW) THz systems [6] and hyperspectral imaging [7] in soil samples. The first results using THz radiation for soil analysis in the range of 258–375 GHz demonstrated a specific interaction with soil particles [8]. In this frequency band, the particle size in the range of a millimeter causes Mie scattering [9,10,11]. Mie scattering, named after the physicist Gustav Mie, is the elastic scattering of electromagnetic waves by particles whose diameter corresponds approximately to the wavelength of the radiation. In the case of multiparticle scattering, Mie scattering shows a complex spectral behavior and varies depending on the local position of the beam. Hyperspectral imaging overcomes the problem of the local variability within the sample and the problem of the interpretation of a single measurement because it combines imagery and spectral behavior. Therefore, most hyperspectral applications and research result from this combination [12,13,14,15,16,17,18,19,20,21,22,23]. The human eye can easily interpret the image and identify different areas, interface regions, and buried objects. Therefore, THz imaging is used for defect or artifact detection [24]. Additionally, critical samples can be simultaneously measured under identical conditions, with fewer time effects caused by the long measuring time. Conversely, the time effects can be compared in a simultaneous measurement, and exchanges between different samples can be analyzed. The measurement of multiple data points helps to overcome individual effects, and common areas can be interpreted statistically. In this way, new images can be created. A disadvantage of this imaging method is the additional complexity of the focus plane. Sharp results can be produced only for features in the focus plane. The high Mie scattering of sand particles prevents beam formation in the material, and a focus plane does not exist under such conditions. This makes generating imagery of soil samples difficult. In order to develop a THz measurement system for a challenging task such as soil characterization, an experimental setup must be developed that allows differentiating and isolating the many different influencing parameters, and at the same time enables an imaging approach by precise localization of each measurement reading within the sample’s dimension. Such an experimental setup will enable basic research on the potential of soil sample characterization through hyperspectral analysis of THz radiation transmission/reflection patterns. This paper describes the development of a THz hyperspectral imaging experimental system for soil sample characterization. With this setup, the authors note the advantages of this method for testing complex samples such as soil samples. This paper discusses the following research questions:

- Can the effect of scattering on image quality be demonstrated by hyperspectral imaging?
- Can the imaging localize artifacts or measurement errors?
- How can the imaging identify homogenous sample regions for statistical comparisons?

## 2. Materials and Methods

Hyperspectral imaging of difficult samples with terahertz radiation is a complex task because of the influence of many physical parameters. It starts with the complicated behavior of the Rayleigh and Mie scattering of thousands of particles. In previous studies, the CELES software [25] enabled the simulation of such complex Mie scattering arrangements and can be used to visualize the results. However, the simulation is not a part of the current work; rather, the measurement possibilities of the presented setup will be demonstrated. The experimental setup described consists of the soil samples and holders, the THz spectrometer, the sample positioning system, the operating software, and the data analysis.

Additionally, natural soil is a complex sample material and soil functionality depends on multiple parameters, including particle size distribution, mineral content, carbon content or organic matter, water, biology, and physical and chemical properties. Therefore, all first measurements were made on simplified soil samples to reduce the number of parameters. The results in this article focus on the imaging possibility of hyperspectral THz measurement.

### 2.1. Soil Samples and Holders

**Figure 1:**
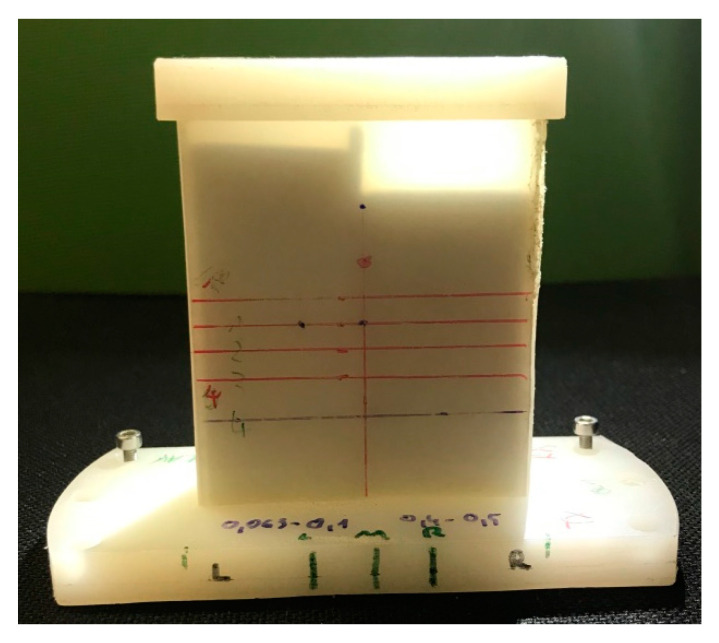
HDPE (high density polyethylene) sample holder for 10 mm sample thickness. A two millimeter thick piece of paper separates the holder in the middle. The left chamber is filled with the 63–100 µm quartz fraction, and the right chamber is filled with 400–500 µm quartz particles.

**Figure 2:**
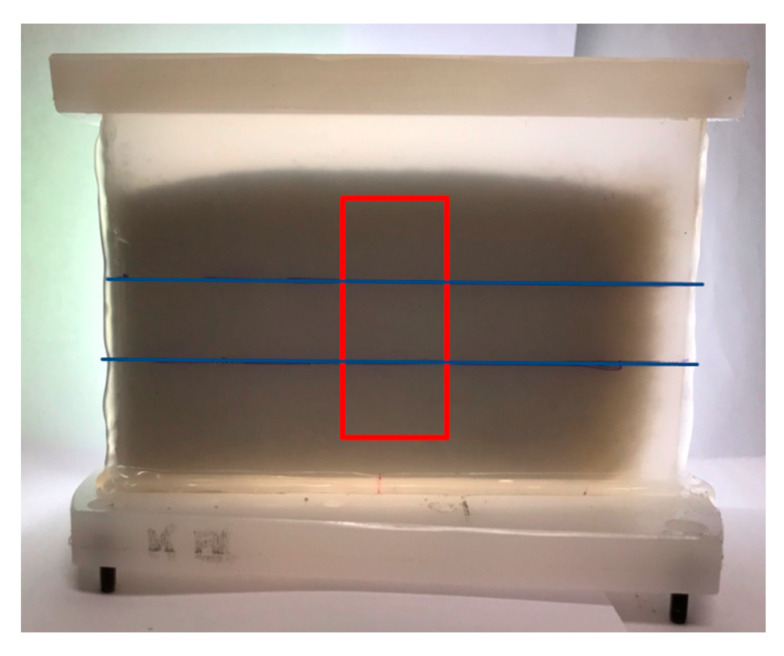
Wedge-shaped sample holder filled with three different soil samples. The blue horizontal lines show the lines between the samples. Samples are quartz sand with different particle sizes. At the bottom is the 100–200 µm fraction, in the middle is the 400–500 µm fraction, and on top is the 500–600 µm fraction. The red rectangle shows the measurement area for this example.

**Figure 3:**
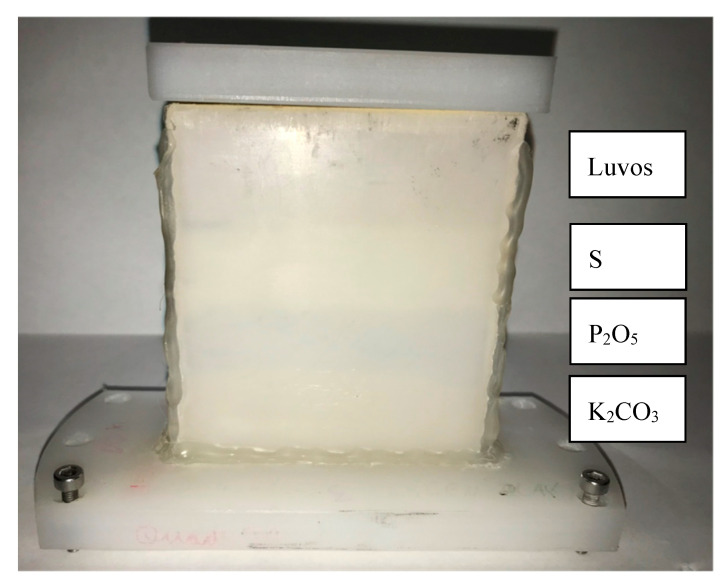
HDPE sample holder for 10 mm sample thickness filled with four different soil samples. The topsoil is pure Luvos^®^ Healing Earth. The next sample is Luvos with 20% sulfur added. The third sample is Luvos with 20% P_2_O_5_, and the bottom sample is Luvos with 20% K_2_CO_3_. All layers are separated with 50 µm thick aluminum foil.

**Figure 4:**
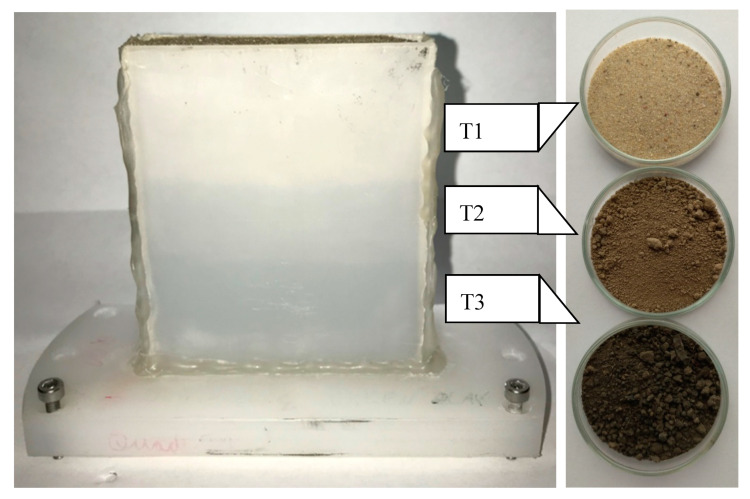
HDPE sample holder for 10 mm sample thickness with three different soil samples. The samples are natural soil samples from the Potsdam region and have different compositions.

The measurement is influenced by the sample holder, the preparation of the sample, the filling of the sample holder, and the compaction (process) in the sample holder. Even the measurement of the empty sample holder is not adequate for calibration because of the different interface situation at the sample holder walls. The best filling substance for calibration is oil [26], but this discussion is not part of this work. The sample holders demonstrated may not be the best solution but rather are a good practical approach. All samples in this study were placed in sample holders made of HDPE (high density polyethylene) with a wall thickness of 2 mm. The holders were parallel, box-shaped, or wedge-shaped. The wedge-shaped sample holder enables simultaneous measurements of different thickness. The sample thickness was addressed accordingly. In some cases, the sample holder was separated into domains with different samples (Figure 1, Figure 2, Figure 3 and Figure 4) to demonstrate the resulting contrast in the corresponding image. Figure 1 shows a box-shaped sample holder with a 10 mm sample thickness. The box was divided with a 2 mm thick piece of paper and filled with quartz particles in different size fractions.

The free configuration of the scan positions helps to reduce measurement times. For a sample preparation as shown in Figure 1, only the interface regions are sampled with smaller step widths for higher resolution. Areas in a homogenous sample region can be represented with a few measurements and can be scanned with a larger step width.

Additionally, the dependency of the sample thickness can be analyzed simultaneously if a wedge-shaped sample holder is used (Figure 2). The sample holder was filled with quartz sand of three different particle sizes. The overlay in Figure 2 with the blue lines indicates the boundary between the three samples. These sample holders have a length-to-thickness ratio of 5:1. The smallest sample holder started at zero thickness, the middle sample holder started at 5 mm, and the largest sample holder started at 10 mm.

Additionally, the sample holder can be filled with different materials, as shown in Figure 3 and Figure 4. Therefore, identical measurement conditions are established, and quantitative differentiation is enabled. For samples that change over time, the fastest scan line across the different materials is selected to minimize the time difference. Every scan pattern for the image can be implemented by the user (see Section 2.4).

Luvos^®^ Healing Earth is a commercial product (Heilerde-Gesellschaft Luvos Just GmbH & Co. KG, Otto-Hahn-Strasse 23, 61,381 Friedrichsdorf, Germany) available in most drugstores. A 100 g quantity of Luvos was prepared twice in four different mixtures containing K_2_CO_3_, P_2_O_5_, S, and MgCO_3_. The LUVOS healing clay with precisely weighed additives was homogenized with a MUK mixer from Fluxana (FLUXANA GmbH & Co. KG, Borschelstr. 3, 47,551 Bedburg-Hau, Germany) for 10 min at 3000 rpm in a mixing cup. Figure 3 shows the maximum concentration of 20% of each additive to generate a contrast in the THz image. 

Figure 4 shows the rectangle sample holder filled with three sieved natural soil samples. All aggregates were broken by hand pestling. The color of the soil sample indicates the amount of organic content. Table 1 shows the associated results.

**Figure 5:**
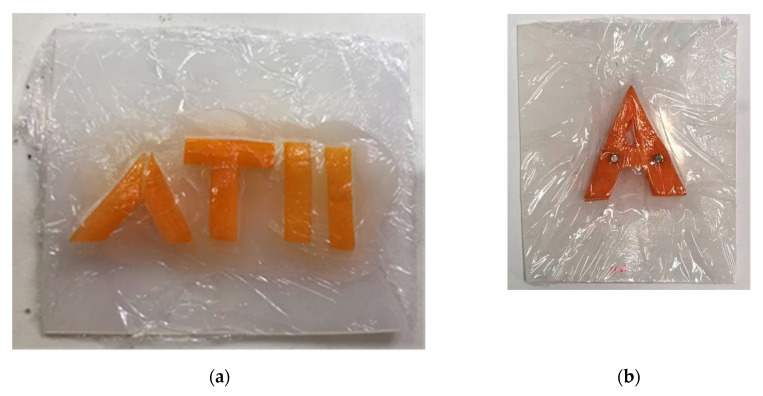
Cut carrot pieces for demonstration: (**a**) carrot pieces forming the letters “ATII”; (**b**) a carrot piece in the shape of the letter “A”.

Buried objects are often used to demonstrate the penetration capability of THz radiation. Organic material such as carrot pieces (Figure 5), for example, in silt or clay, is detectable, but detection is not possible in sand because of the strong Mie scattering. With hyperspectral imaging, all images at the different frequencies can be averaged, and the mean damping can be estimated. Additionally, the focus is adjusted to the middle position of the sample holder and thereby to the surface of the carrot. Therefore, the interface region between the carrot and the scattering sand is addressed, but more scattering will avoid a sharp focus. The interface region tends to result in more scattering if addressed with a sharp focus.

**Figure 6:**
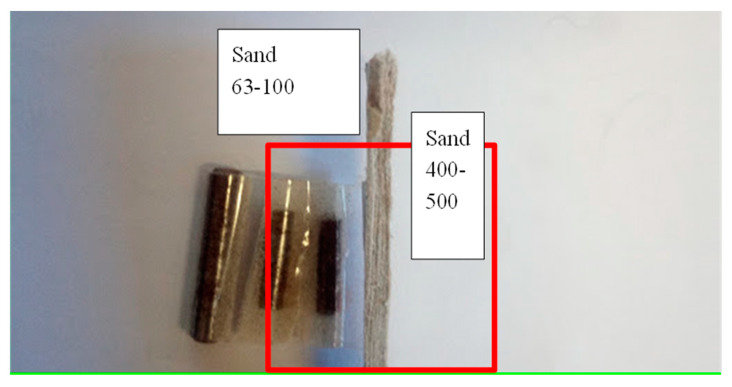
Additional filling of the sample holder in Figure 1. Three steel tubes were fixed with tape and buried in quartz sand. The left side was filled with the 0.063–0.1 mm fraction, and the right side was filled with the 0.4–0.5 mm fraction. The divider is 2 mm thick paper.

The carrot samples in Figure 5 were placed in the sample holder, and the sample was filled with quartz material in different particle fractions. The next example in Figure 6 shows a setup with metal pieces buried in the 10 mm sample holder. The imaging is demonstrated in this paper, but the complicated task of detecting conducting materials and their surface waves is not described in detail. 

### 2.2. THz Spectrometer

**Figure 7:**
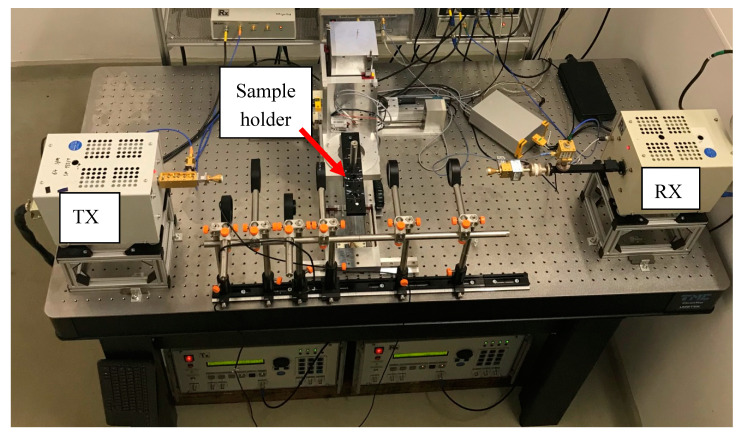
THz spectrometer setup on an optical damping table. TX is the transmitter setup, and RX is the receiver setup.

The THz spectrometer in Figure 7 is the same as that described by Dworak et al. [8], and the full electronic setup is described in detail. The system has an emitter and a receiver, and sweeps from 258 to 375 GHz. The applied distance between each spectral measurement point is adjustable, and the practical range is from 0.3 to 0.01 GHz. The system consists of two backward wave oscillator tubes, which generate the main RF power. The frequencies are up converted with a frequency doubler and tripler. The receiver side follows the frequency sweep of the emitter side with an offset of 300.5 MHz. Both signals are multiplied in a second harmonic mixer and down converted to 905 MHz. An additional down conversion results in a 1.5 MHz measurement signal. The consequent AC measurement establishes a 1/f noise free sample signal and the high dynamic range of the system. The dynamic range of the system is about 100 dB.

The spectrometer includes an optical system that focuses on the image plane and collects the THz radiation. The TPX lenses are from TYDEX^®^ (Kavalergardskaya str. 16, 191,015 St. Petersburg, Russia).

### 2.3. Sample Positioning

**Figure 8:**
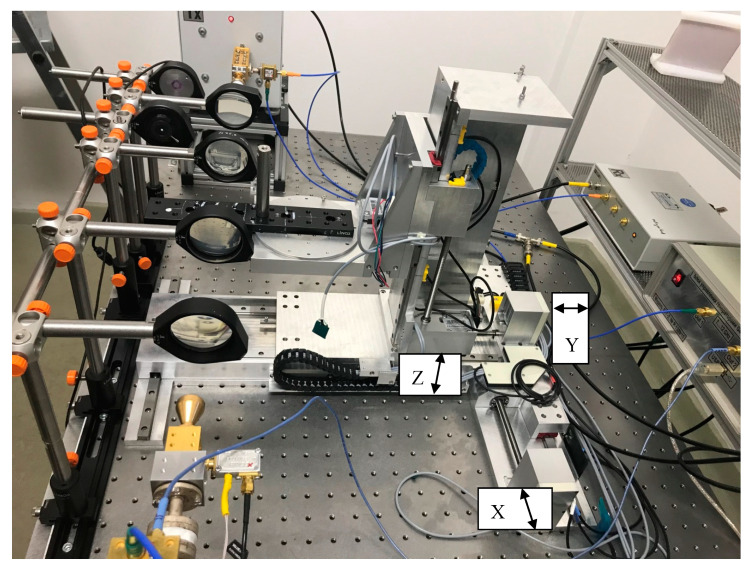
Image of the hardware setup. The optical setup of the THz beam is on the left side. The positioning table is in the center.

The sample positioning setup in Figure 8 is custom made and consists of four motorized axes. The axes can move ±8 cm in the XYZ directions, and the rotation axis can move ±360° in the Z direction. The minimal step width in micro step mode 32 is 0.4 nm, and the minimal step width allowed by the self-developed control software is 10 µm. The minimal rotational step width in micro step mode 32 is 0.005625°. Each axis motor has its own microcontroller, which are connected to a master controller. The master is connected to a PC. The PC runs the self-developed operating software, sends the position commands to the master, and waits for the “done” response.

### 2.4. Operating Software

**Figure 9:**
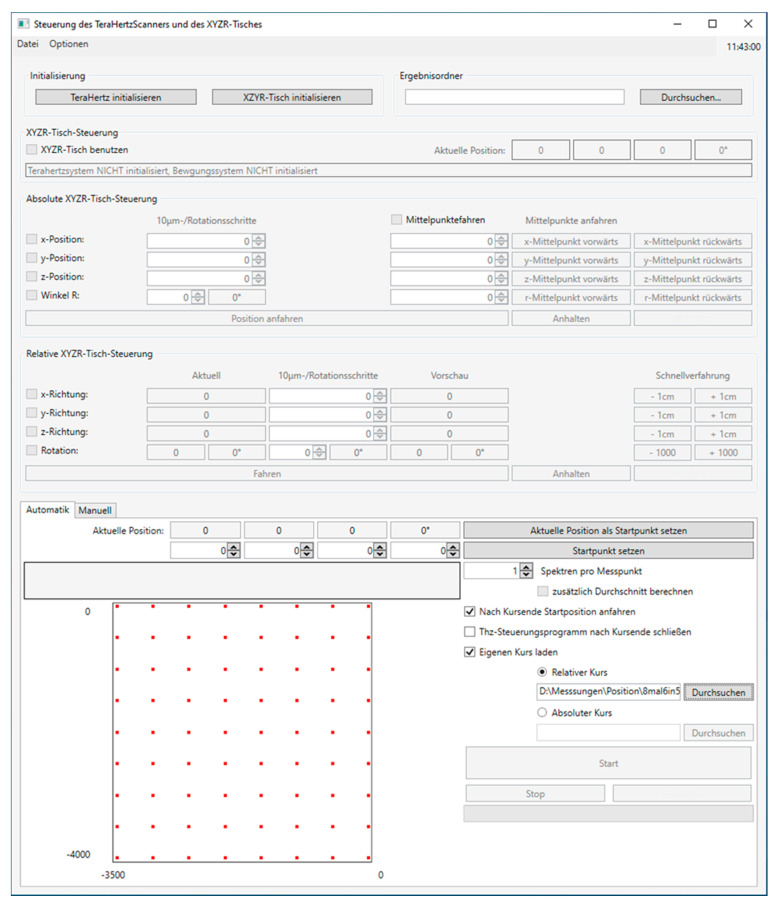
Control software for the motorized sample positioning table. Measurement points are established by an external table. The software automatically starts with the selected number of spectral scans at each local measurement point.

The operating software consists of two parts. The first part is the THz scanner control software from ELVA-1, St. Petersburg, Russia. The second part is the self-developed user GUI (Figure 9) that combines the scanner software with the positioning control. The user can navigate to a dedicated measuring position or can load an Excel spreadsheet with a list of positions. The software will move the sample to the positions in the order of the list and perform the preset number of measurements at each position. The list of positions is externally set and loaded through the software; in this way, any scan pattern can be generated.

### 2.5. Data Analysis

**Figure 10:**
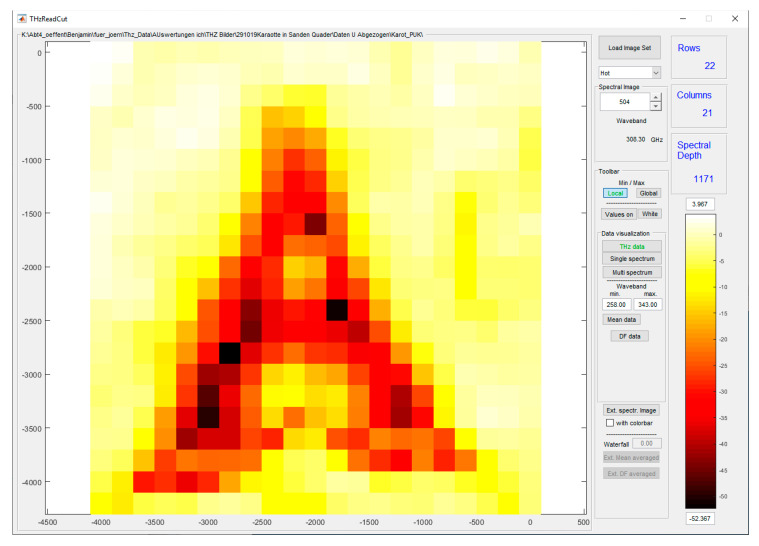
MATLAB GUI for the hyperspectral image display. Example of the control menu, which can be adapted for adequate evaluation.

The third part of the software is a self-developed MATLAB^®^ GUI for visualizing the spectral cube and performing the data analysis. The image in Figure 10 displays the amplitude of a selected frequency. Clicking on an image pixel with the mouse opens the spectral plot at this measurement point. Additional data analyses, such as the mean values, dynamic factors, and spike counts, can be displayed. The MATLAB environment allows the possibility of implementing any conceivable analysis.

The MATLAB GUI in Figure 10 shows 22 rows and 21 columns for this example, but there are no restrictions in any direction. Additionally, new functionalities can be developed and run by adding new buttons.

### 2.6. Dynamic Factor

The MATLAB GUI can perform all kinds of analysis that can be designed in MATLAB. One example of this capability is the “dynamic factor” (DF). Soil samples with high Mie scattering show highly dynamic behavior in the spectrum [8]. The “dynamic factor” describes this behavior, where DF is the dynamic factor, i is the index, and A(f_i_) is the amplitude of the transmitted signal at the specific frequency i in the spectrum:

(1) $$\mathrm{DF}=\frac{1}{i_{\mathrm{EndFreq}}-i_{\mathrm{StartFreq}}}\sum_{i_{\mathrm{StartFreq}}+1}^{i_{\mathrm{StartFreq}}}|A(f_{i})-A(f_{i-1})|$$

DF only represents the sum of the difference between neighbors; therefore, it is less sensitive to the general shape or tendency of the spectrum.

### 2.7. Spatial Resolution

**Figure 11:**
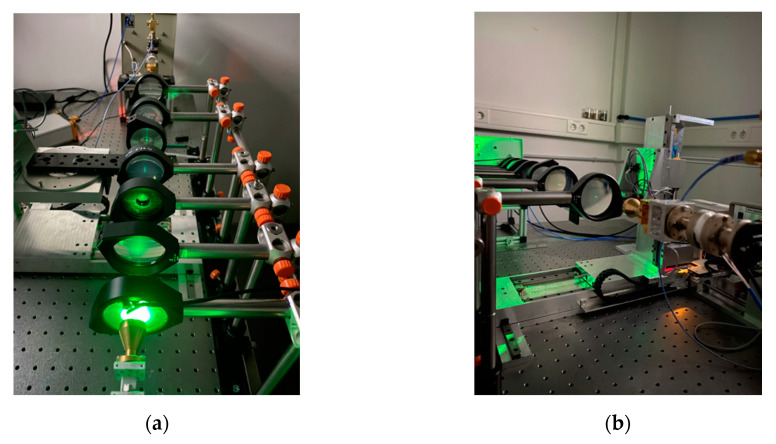
Green LED light enables axis adjustment: (**a**) view from the incoming side; (**b**) view from the outgoing side with a pinhole sample.

The optical setup shown in Figure 8 and Figure 9 focuses the beam on the sample layer. The spatial resolution can be analyzed by moving dedicated samples through the beam. The resulting damping of the signal amplitude represents the convolution of the beam shape and the sample geometry. The knowledge of the sample shape enables the deconvolution of the measured signal and the beam shape can be generated. A common approach is to use a small aperture “pinhole” as the sample. Measurements with a hole diameter of one millimeter are performed in two planes. The first plane is the image plane, and the second plane is along the beam axis. To reduce the search and scan time required to find the pinhole with the beam, the setup was preadjusted with green LED light (Figure 11).

## 3. Results

The results demonstrate the imaging possibilities of the described setup. The hyperspectral results always consist of a full spectrum for each image pixel. Therefore, each frequency step has its own image. Not all images are shown, and just one image of one frequency step is provided. More often, processed image data such as the mean values or DF are shown.

### 3.1. Characterization of the Setup

**Figure 12:**
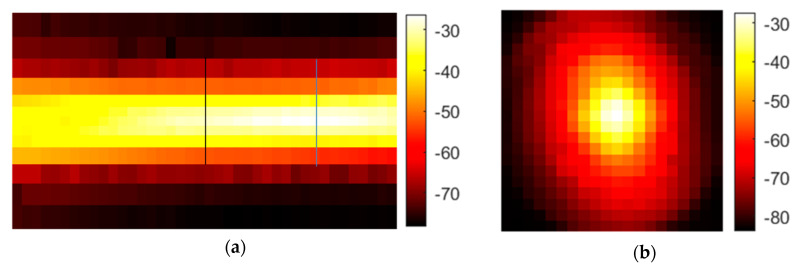
Measurement of the transmitted signal intensity through a 1 mm pinhole aperture on a dB scale: (**a**) Mean intensity along the beam axis. The black line represents the expected focus plane, and the blue line represents the real focus plane. (**b**) Mean intensity orthogonal to the beam axis in the focus plane.

The characterization of the optical performance of the THz setup demonstrates that the behavior is similar to that in optical systems with visible light but with lower resolution. The in-axis measurement with the 1 mm aperture (pinhole) in Figure 12a shows the typical waist of a lens-focused beam. The waist arises due to the imperfections of the source geometry and the imperfections in the lenses.

The image of the focus plane in Figure 12b shows the typical Gaussian shape of the intensity. The pixel size is 1 mm^2^, and the focus is acceptably good, especially with respect to the waveguide and the horn antenna as the source emitter.

**Figure 13:**
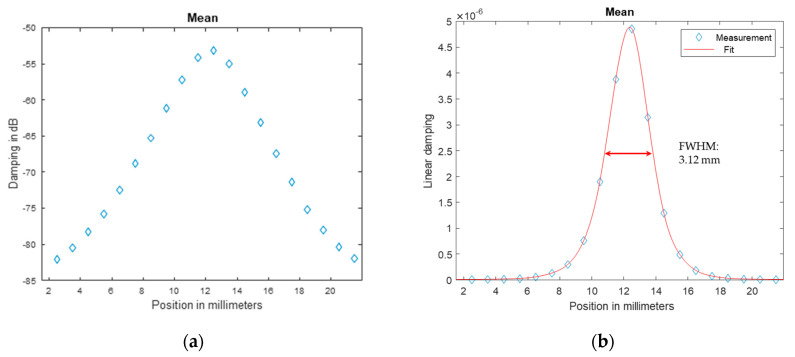
Intensity plot of the central line: (**a**) the full width at half maximum (FWHM, −67.5 dB) is 10 mm on the dB scale; (**b**) the FWHM is approximately 3 mm on the linear scale.

Figure 13 shows the Gaussian profile in one dimension, and subtraction of the aperture diameter of one millimeter from the FWHM results in a resolution of approximately 9 mm on the dB scale. On the linear scale, the half-height is −55.5 dB, and the resulting resolution is approximately 2 mm. Therefore, all images are blurred in that range. Considering the setup, this is a good resolution, but it is four times worse than the theoretical Abbe resolution of 0.5 mm at 1 mm wavelength with maximum aperture angle. Additionally, all of the following images are displayed on the logarithmic scale to enhance visibility. Otherwise, the high dynamic range would result in only black and white images. Often, automatic scaling of the image colors is activated. This must be taken into account when comparing the colors. If the comparison is important, absolute scaling is applied.

### 3.2. Simultaneous Measurement of Multiple Samples

The simultaneous measurement of multiple samples has several benefits. First, the direct contrast between the samples in the image gives a good overview of the image and locates the regions of the different materials in the image. Areas of artifacts can also be identified. Second, changes to the measurement setup are typically avoided in simultaneous measurement. Even small changes in the frequency response could induce incorrect results if the spectral differences between somewhat similar samples were analyzed. In particular, the DF of a low-scattering material is highly affected by these small changes. Additionally, the scattering of interface regions is higher than that of bulk material.

**Figure 14:**
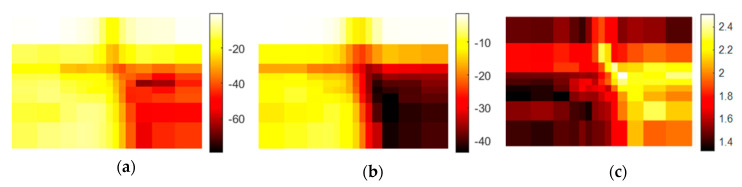
Images resulting from the setup in Figure 1 are shown. Different step widths between the measurement pixels were applied. The transmitted signals were recorded: (**a**) amplitude in dB of the center frequency of 316.5 GHz; (**b**) mean value in dB of the spectral range from 258 to 343 GHz; (**c**) dynamic factor (DF) for the same spectral range.

The mean (damping) values of the 100–200 µm and 400–500 µm particle size fractions in Figure 14 are −11 and –43 dB, respectively. It is clear that the interface regions vary due to the beam divergence and the frequency-dependent scattering behavior indicated by the DF. The comparison of the mean values and the DF indicates that the 400–500 µm fraction shows much higher scattering than the 100–200 µm fraction. This could be explained by Mie scattering [10].

**Figure 15:**
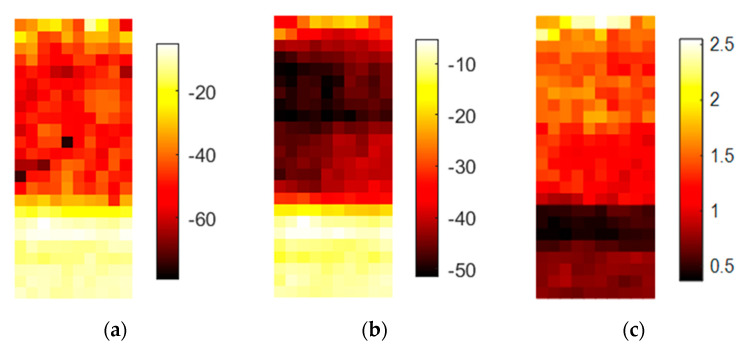
This figure shows the results for the setup in Figure 2. Samples are quartz sand with different particle sizes. At the bottom is the 100–200 µm fraction, in the middle is the 400–500 µm fraction, and on top is the 500–600 µm fraction: (**a**) amplitude in dB of the center frequency of 316.5 GHz; (**b**) mean value in dB of the spectral range from 258 to 343 GHz; (**c**) DF for the same spectral range.

Mie scattering depends on the frequency and local composition of the particles in the sample. Figure 15a demonstrates that at a fixed frequency the geometrical position changes the amplitude depending on the random arrangement of the particles, which is homogeneous in the far field. The minima and maxima change positions at other frequencies. Hyperspectral imaging helps to explain this situation. Additionally, the mean value and the DF in Figure 15b,c show the principal differences among the three particle fractions. Going from low to high particle size, mean damping values around 12, 40, and 50 dB are observed; the DF also increases, with values around 0.7, 1.3, and 1.5. The higher scattering causes more damping and more dynamic behavior, and even the small difference between 400–500 µm and 500–600 µm is precisely indicated. Before analyzing the spectral information in depth, it is important to know the sample position at which the spectrum will be collected.

**Figure 16:**
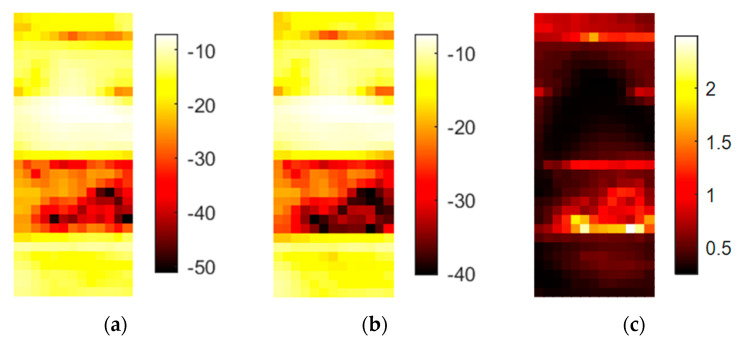
This figure shows the results for the setup in Figure 3. The first two samples are Luvos and Luvos with 20% sulfur added. The third sample is Luvos with 20% P_2_O_5_, and the bottom sample is Luvos with 20% K_2_CO_3_: (**a**) amplitude of the center frequency of 316.5 GHz; (**b**) mean value of the spectral range from 258 to 343 GHz; (**c**) DF for the same spectral range.

The images in Figure 16 show that some artifacts can exist, and it is important not to use these areas in the spectral analysis.

**Figure 17:**
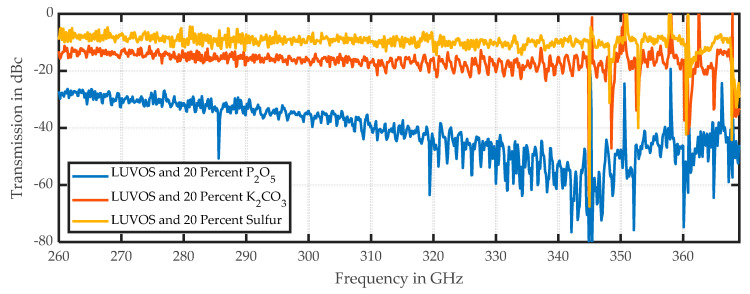
Spectral plot of the Luvos sample with three different dopings. Each spectrum represents one pixel from the respective doped LUVOS fraction.

An investigation of this artifact is not part of this work, but appears to be an artifact of preparation. The high concentration of phosphorus in sample 3 induces a tendency to agglutinate. Therefore, it could be possible that the higher DF value indicates the shape of an agglomerate. Furthermore, the interface heights are addressed by the DF. However, it is still precisely indicated in the image, which demonstrates the advantages of hyperspectral imaging. Additionally, the difference in the spectral amplitudes in Figure 16 is not analyzed here. To speculate, doping the same matrix with different chemicals could cause changes in the hygroscopic/hydrophobic behavior of the material; higher or lower amounts of water could be causing the effect. Figure 17 shows the spectral behavior of the three doped samples. 

The sulfur and the potassium samples (yellow and red lines) are offset by approximately 7 dB, which could have been induced by their different water levels. The phosphorus sample (blue line) shows nonlinear behavior, and the highest damping occurs in the range between 343 and 347 GHz. This cannot be explained only by the presence of more water and must be analyzed in future studies. Nevertheless, hyperspectral imaging enables the detection of complex behaviors.

**Figure 18:**
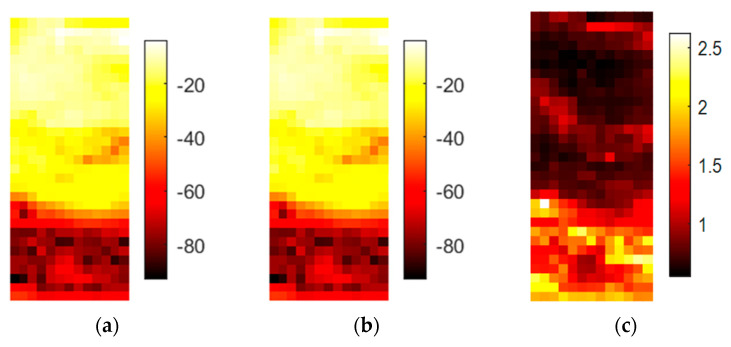
This figure shows the results for the setup in Figure 4. Samples are natural soils: (**a**) amplitude in dB of the center frequency of 316.5 GHz; (**b**) mean value in dB of the spectral range from 258 to 343 GHz; (**c**) DF for the same spectral range.

**Figure 19:**
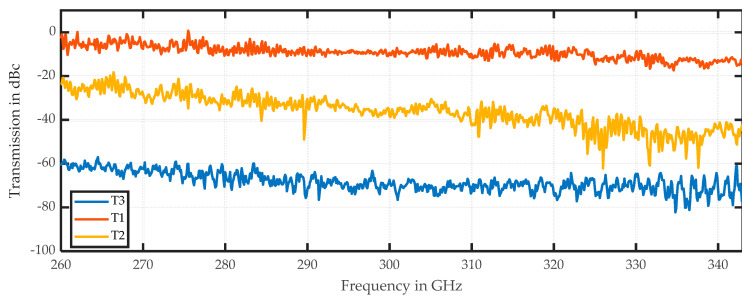
Spectral plot of the three soil samples. T3 is the bottom sample with the most organic content. T2 is the sample in the middle. Each spectrum represents one pixel from the respective soil fraction.

The three different soil samples in Figure 18 show different damping and scattering patterns, and their typical spectral responses are displayed in Figure 19. The scattering amplitudes vary by the local position, and the interface region cannot be precisely localized by the DF. 

The main difference among these soil samples is the damping-related offset in the spectrum. Sample T2 also has a higher slope than the other samples and some variation at approximately 300 GHz. A similar slope for sample T3 stops at approximately 290 GHz and is then horizontal. Damping values of soil T3 with the most organic content are 50 dB higher than those for soil T1 that is dominated by quartz sand. The results are not remarkable, but hyperspectral imaging enables the comparison of spectral features in addition to the visual differentiation of multiple samples in one run. 

### 3.3. Measurement of Buried Objects

THz imaging is well known for its use in detecting buried objects. Detecting buried objects in soil samples is much more difficult than detecting buried objects in other substrates because of the scattering of sand particles. This scattering disorders the focus of the beam, and a sharp image of the objects cannot be obtained.

**Figure 20:**
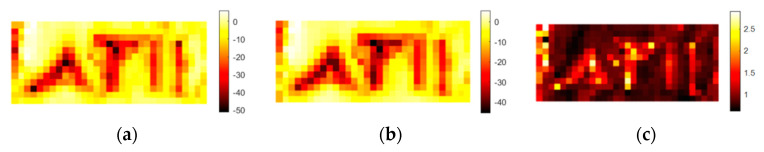
This figure shows the results for the setup in Figure 5a. Three carrot letters are buried under quartz clay: (**a**) amplitude in dB of the center frequency of 316.5 GHz; (**b**) mean value in dB of the spectral range from 258 to 343 GHz; (**c**) DF for the same spectral range.

**Figure 21:**
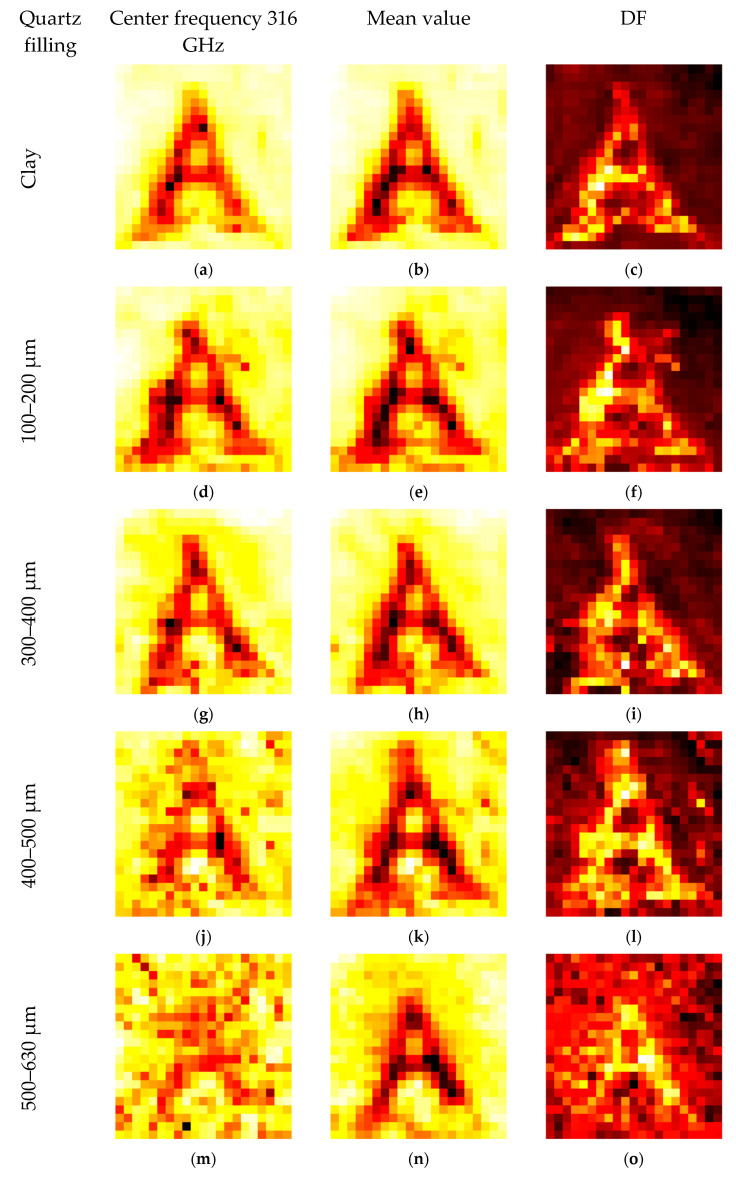

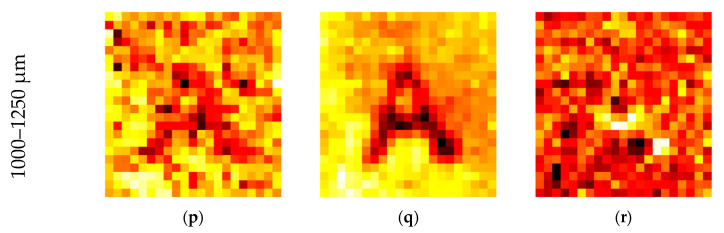
The letter in Figure 5b was buried under different quartz sand fractions. The size of the fractions increases in every row: (**a**,**d**,**g**,**j**,**m**,**p**) amplitude of the center frequency of 312.5 GHz; (**b**,**e**,**h**,**k**,**n**,**q**) mean value of the spectral range from 258 to 343 GHz; (**c**,**f**,**i**,**l**,**o**,**r**) DF for the same spectral range.

Figure 20 shows the high absorption of water by the carrot letters. The image is blurred, and some artifacts are visible around the middle letter “T” but, to the human eye, the letters are still readable. The distortion in the image depends directly on the scattering behavior of the matrix. The demonstration of this effect is shown in Figure 21 with the test letter “A” from Figure 5b. The sample holder is consequently filled with larger sand particles, and therefore, the scattering also increases. Here, the defocusing also increases and, unlike in the case with clay, the interface between carrot and sand cannot be addressed.

**Figure 22:**
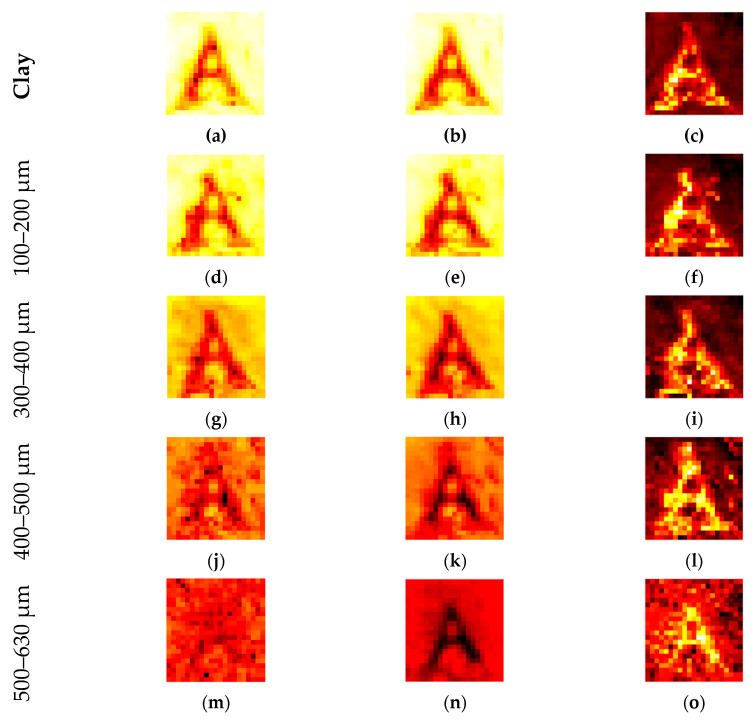

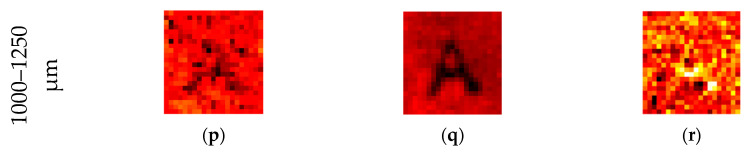
The same images from Figure 21, but with a common scale for the frequency of 316.5 GHz and the mean value.

Figure 21 demonstrates the effect of image distortion caused by the scattering matrixes. An artifact is detected in d, f, and g. Regarding the previous artifacts, sample preparation caused this effect, which is not visible in the other images. It is notable that the letter is not visible in Figure 21m,p, but the corresponding mean values clearly show the “A”. The average of the high number of images filters out the general higher damping of the carrot. However, averaging is not always a sufficient filter. Without autoscaling and a common color bar for all images, the general tendency toward higher damping in the larger sand fractions can be noted. Figure 22 is identical to Figure 21, except for the common scale and the size of the images.

**Figure 23:**
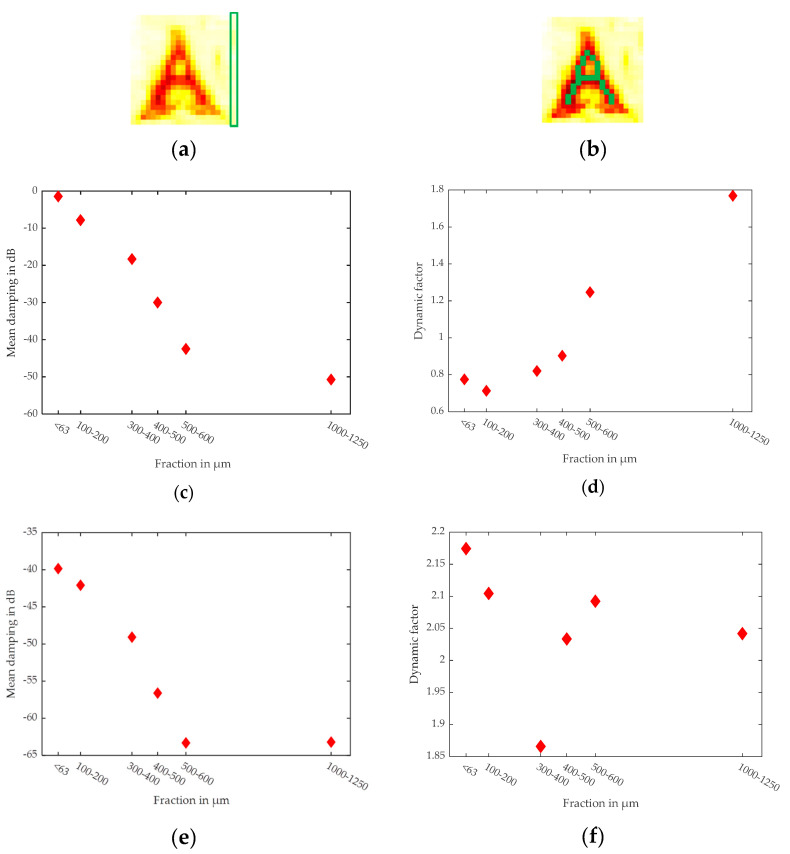
Average of the right side vertical pixel lines plotted over the particle size: (**a**) pixel area for bulk analysis; (**b**) green pixels indicate used pixels for carrot area; (**c**) mean values (in the middle range the slope is about 10 dB/100 µm); (**d**) DF of the bulk material; (**e**) mean values of carrot pixels; (**f**) DF of the carrot pixels.

The logarithmic scale still enables the “A” to be visible in the mean images. The values of all pixels in the vertical right corner line of fraction dependent images in Figure 22 were averaged to obtain the common values of the quartz particle size dependent DF and mean values (damping) (Figure 23a). The green pixels in Figure 23b indicate the used pixels for the analysis of the carrot area.

Figure 23 shows the particle size dependent mean (damping) values and the DF. The slope of the bulk material in the middle fraction is about 10 dB/100 µm for the mean values in Figure 23c. This demonstrates a high sensitivity for the particle size with this method. The scattering of the 1–1.25 mm particles is influenced by the saturation of the Mie scattering at about πD/λ, where D represents the particle diameter and λ the respective wavelength used for measuring. Therefore, the mean value is less reduced. The DF follows a different trend, and both values are good candidates for machine learning inputs. The reduced scattering of the 1–1.25 mm fraction also causes a reduced DF for that fraction. The situation is even more complicated for the carrot area. The beam focus at the interface region induces higher scattering. Additionally, the water damping of the carrot reduces the signals of about 30 dB. Therefore, the signal damping influences more fractions and the slope of the mean values is reduced to 7.5 dB/100 µm. This damping reduces the scatter amplitudes and the higher defocusing of lager particles causes the resulting DF to be more or less constant. The interface region induces higher scattering, but the water damping and the defocusing reduces this effect. 

**Figure 24:**
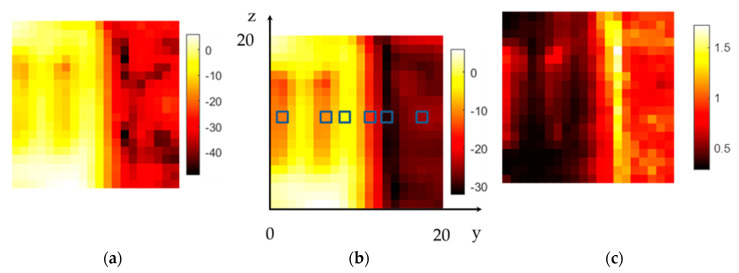
The result of the setup in Figure 6. The left side was filled with the 0.063–0.1 mm fraction, and the right side was filled with the 0.4–0.5 mm fraction. The two buried steel tubes are on the left side of the images: (**a**) amplitude in dB of the center frequency of 316.5 GHz; (**b**) mean value in dB of the spectral range from 258 to 343 GHz; the blue squares indicate the pixel position for the example spectrum; (**c**) DF for the same spectral range.

Conductive materials such as metal cylinders are more difficult to detect, as expected. The electromagnetic wave can travel around the surface of the cylinder, and the cylinder appears to be transparent. Figure 24 shows the results of the experiment in Figure 6. Two steel tubes were buried under quartz sand fractions from 63 to 100 µm.

**Figure 25:**
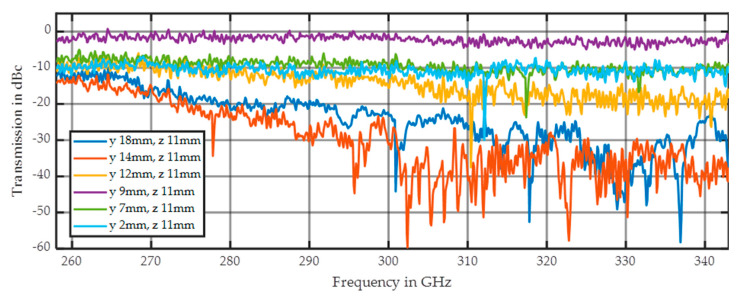
This figure shows the five spectra indicated in Figure 24.

Both tubes are visible, but their influence is even smaller than the scattering of the 400–500 µm fraction of quartz sand on the right side of the image. Additionally, the interface region between the paper and the larger fraction shows notably more scattering. This is also indicated by the average DF of 1.5 in this area, which is significantly higher than the average DF values of 0.7 and 1.0 for the small and big particle fractions, respectively, in the sample holder. Figure 25 shows the resulting spectral response at the dedicated positions. Both tubes (at y = 2 and 7 mm) show somewhat similar spectral behavior to the small sand fraction (at y = 9 mm) but with an offset of 10 dB in additional damping. Additionally, the increased scattering behavior in the interface region (at y = 14 mm) is also visible in the spectral response indicated by the highest average damping value of about 30 dB.

## 4. Discussion

THz hyperspectral imaging enables transmission mode analyses of difficult samples, but is strongly influenced by Mie scattering. Thus, all of the scattering along the beam pathway disorders the beam and causes image distortion. Therefore, the imaging of soil samples is extremely difficult and not possible if the sample is dominated by large sand particles of approximately 1 mm. Conversely, the amount of scattering indicates the particle size fraction and could be an important measurement tool for soil samples. The analysis of the setup demonstrates a local resolution of approximately 2 mm, which is reasonably good for an emitter with a wave guide and horn antenna. Therefore, even images with smaller pixel sizes are blurred in that range. However, with respect to the soil samples, scattering is the dominant factor that causes image distortion. Looking at image “p” in Figure 21, the scattering blurs the image of the carrot letter “A”. The image looks like noise, but is not noise; it is reproducible scattering. The multiple images for each frequency step enable statistical analysis in the MATLAB environment. For example, the image of the mean value clearly reproduces the letter “A”.

The imaging helps the user to identify local artifacts and therefore avoid taking these pixels into account in further statistical analyses of this sample region. For example, the mechanical stress on the carrot can lead to water leakage. The artifacts are mainly caused by sample preparation errors, but measurement failures with missing signal amplitudes and interface regions with different behaviors can also be identified, if they happen or exist. This makes the hyperspectral setup a great measurement tool for critical and nonhomogeneous samples, and reduces fault analysis and interpretations. Additionally, because unwanted areas are taken out of consideration, the pixels can be classified, and common sample areas can be combined for further analysis in the MATLAB environment, for example. This analysis could reveal a typical spectral response or indicate that a more complex situation exists. Furthermore, the statistically confirmed results can be compared with the simulation results in the future. Nevertheless, the visualization of the spectral result often enables a quick interpretation of the situation; for example, water added the offset in the frequency range. This does not mean that a single frequency is sufficient for analyzing this behavior because scattering still dominates the amplitude of each frequency.

## 5. Conclusions

THz hyperspectral imaging provides all of the advantages of normal light hyperspectral imaging but enables transmission mode analyses of difficult samples. Here the transillumination of cm thick samples is possible, and the optical resolution could be up to 2 mm. The energy of THz radiation is not high enough to change the energetic states of atoms or molecules, but the physical scattering and dielectric constant still produce contrasts in the THz images. Organic content in soil samples could add about 50 dB signal damping, as demonstrated in Figure 19. The reduced resolution compared to optical measurements does not enable the analysis of single particles in an aggregation, but enables measurements in the volume. The average of multiple pixels generates a common result of a sample. Figure 23 demonstrates the sensitivity of the setup for particle size of about 10 dB/100 µm for quartz sand. Additionally, the results for the mean value and the DF in Figure 23 show different trends for the particle size and could be good input candidates for future machine learning approaches. Additionally, both parameters are influenced by water damping of organic material such as carrot. Therefore, THz hyperspectral imaging is a valuable tool for analyzing difficult samples such as soil samples.

## Figures and Tables

**Table 1:** Analysis of the air-dried soil samples. The difference to 100% is the remainder, which is so-called “mineral ashes”.

Sample Name	DM105	OM	C	N	S
	%	%	%	%	%
T1	99.97	0.238	0.010	0.001	0.006
T2	99.75	1.554	0.451	0.017	0.030
T3	93.50	30.50	15.50	0.142	3.61

DM 105 is the dry matter of the sample after oven-drying for 24 h at 105 °C; OM is the amount of organic matter; C, N, and S are the concentrations of carbon, nitrogen, and sulfur, respectively.

## Abbreviations

The following abbreviations are used in this manuscript:

Vis-NIR	Visible to Near-Infrared
DF	Dynamic factor
HDPE	high-density polyethylene
THz	Terahertz
GHz	Gigahertz
TX	Transmitter
RX	Receiver
GUI	Graphical user interface
CW	Continuous wave
PC	Personal computer
FWHM	Full width half maximum
